# Behind the Scenes on Hospital Sunday

**Published:** 1920-06-26

**Authors:** 


### Hospital Sunday, June 27, 1920.

### SPECIAL HOSPITAL SUNDAY FUND SECTION.

### BEHIND THE SCENES ON HOSPITAL SUNDAY.

How many of those attending Hospital Sunday services really know the facts? How many congregations understand what Hospital Sunday means?

Since 1873, when the Fund was instituted, one Sunday every year has been devoted to an explanation of the work of the voluntary hospitals, because Canon Miller, to whom the original idea was due, saw that voluntary work for the sick is a Christian duty and desired to revive the association between churches and hospitals which had once distinguished both. In the fifty years which have all but passed since then the Fund has reached an average of £40,000 a year from collections in churches in and around London, and approximately the like amount accrues from the bequests and donations which certain generous people have given to it. The main point, however, is that the dead have given as much, if not more, than the living; for the church collections have rarely passed £40,000, and this means, first, that the Fund has done much of its work by the aid of a few rich people (the late Mr. George Herring was a munificent benefactor); and, second, that the number of contributing congregations does not grow, nor do the churches in the heart of London compare well with those in the home counties.

In the City a small sub-committee of the Fund this year is trying to improve matters. The facts are these. Last year the Bishop of London and a few other church and City dignitaries, invited the City clergy to explain the objects of Hospital Sunday to their parishioners. Six responded. Consequently a new plan must be tried. In the West-end indifference is also conspicuous. The Hospital Sunday Fund is regularly encouraged by the familiar tale of mansions converted into boarding-houses, changes in the financial status of once fashionable parishes, and so forth. But one parish does not decline socially without another parish improving its status; and of these lucky parishes very little is heard. The tide of fashion is now ebbing from the West eastwards. If Cavendish Square is becoming less fashionable, Bloomsbury and Bedford Square are renewing their distinction. From Bloomsbury, therefore, good news is due. But the clergy themselves are often indifferent. They forget that one of their number founded Hospital Sunday; and if they remembered, might perhaps thank little the man whose idea of one Sunday devoted to charity has inspired so many others to impose on the clergy similar claims, so that incumbents are tempted to feel that their congregations are expected to subscribe to everything except their own parish. We allow for this resentment, but point out that Hospital Sunday, for reasons explained already, is in a superior category.

Laymen, many of whom devote their leisure time to hospitals, should ask their minister, when a collection is held on Hospital Sunday, but no sermon is preached upon the text for which it stands, the reason for this omission. They can further remind him that custom allows a minister to be relieved of this task, and that Mr. Arnold James, or some other officer of the Sunday Fund, can probably suggest a medical man or a hospital worker who will give an address to any congregation whose minister is unequal to the chance.

For, it must be repeated, the clergy are growing suspicious of all charitable funds appealing for church support, and do not distinguish between the true and the spurious claimants. The Sunday Fund is often left without the support of the Sunday preacher. Who deserves the blame? There can be, we fear, but one answer. Consequently, the layman must come forward to shoulder a task which the clergy are neglecting. Any official of the Fund will seem to the clergy perhaps an interested party. But there is a great opportunity for a layman of standing who knows what the voluntary hospital secures to the patients, to step out of his way and infect the clergy with his own enthusiasm. If that is done, all the excuses of high taxation and uncertainty in regard to the action of the Ministry of Health will collapse like a house of cards. The cinema does not suffer from high taxation; even the amusement tax has not curtailed its audiences. The motor-car still runs in spite of the cost of petrol. The Derby happily flourishes though people have "no money to spare." Nor are these facts mysterious or alarming. People always find money and time for that which appeals to their sense of importance or to their desire for fun. But the cinema is so simple an expression of this that it needs no explanation. The appeal of sickness, hardly less simple, is yet the result of a primary *recoil* from it. People do not, and ought not, to invite its coming. But beside the Pharisee in us, who likes to turn away, there is the Good Samaritan in us who wishes to help. It is this instinct which the voluntary system of hospital support expresses in bricks and mortar, labour and love; and it is the religious basis of this instinct which Hospital Sunday fosters. For it is not only sympathy, but cheerfulness which the hospitals radiate, because such work for others is the best thanksgiving for *health* which each of us can make. This is the basis of the sermons which should be preached on Hospital Sunday, for unless the motive is there the generosity will be lacking. Will not the reader respond to the call?

